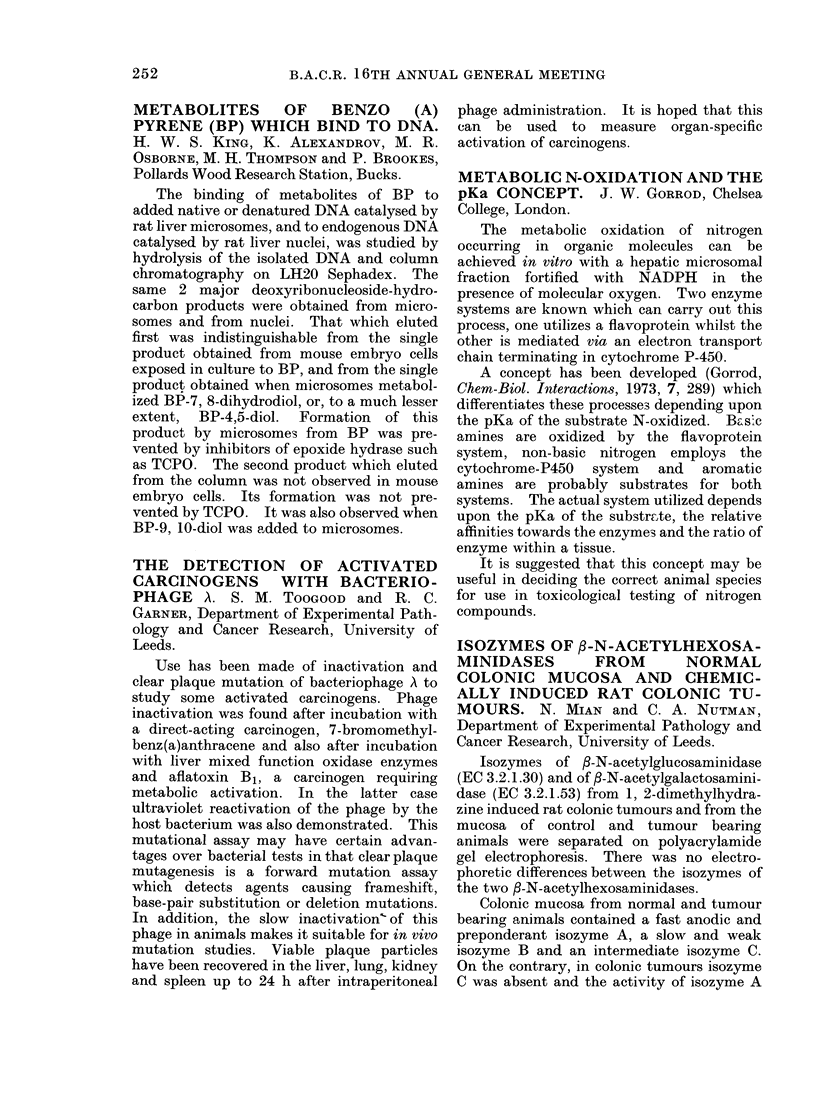# Proceedings: Metabolites of benzo (A) pyrene (BP) which bind to DNA.

**DOI:** 10.1038/bjc.1975.194

**Published:** 1975-08

**Authors:** H. W. King, K. Alexandrov, M. R. Osborne, M. H. Thompson, P. Brookes


**METABOLITES OF BENZO (A) PYRENE (BP) WHICH BIND TO DNA.** H. W. S. King, K. Alexandrov, M. R. Osborne, M. H. Thompson and P. Brookes, Pollards Wood Research Station, Bucks.

The binding of metabolites of BP to added native or denatured DNA catalysed by rat liver microsomes, and to endogenous DNA catalysed by rat liver nuclei, was studied by hydrolysis of the isolated DNA and column chromatography on LH20 Sephadex. The same 2 major deoxyribonucleoside-hydrocarbon products were obtained from microsomes and from nuclei. That which eluted first was indistinguishable from the single product obtained from mouse embryo cells exposed in culture to BP, and from the single product obtained when microsomes metabolized BP-7, 8-dihydrodiol, or, to a much lesser extent, BP-4,5-diol. Formation of this product by microsomes from BP was prevented by inhibitors of epoxide hydrase such as TCPO. The second product which eluted from the column was not observed in mouse embryo cells. Its formation was not prevented by TCPO. It was also observed when BP-9, 10-diol was added to microsomes.